# Actin-Interacting Amphidinolides: Syntheses and Mechanisms of Action of Amphidinolides X, J, and K

**DOI:** 10.3390/molecules28135249

**Published:** 2023-07-06

**Authors:** Anna M. Costa

**Affiliations:** 1 Organic Chemistry Section, Department of Inorganic and Organic Chemistry, Facultat de Química, Universitat de Barcelona, Diagonal 645, 08028 Barcelona, Catalonia, Spain; amcosta@ub.edu; 2 Institut de Biomedicina de la Universitat de Barcelona (IBUB), 08028 Barcelona, Catalonia, Spain

**Keywords:** G-actin/F-actin equilibrium, actin stabilizers and destabilizers, total synthesis, amphidinolides

## Abstract

Amphidinolides are a family of more than forty macrolides of varying sizes and complex structures isolated from dinoflagellates of the genus *Amphidinium*. Although all of them display potent-to-moderate cytotoxicity, their full bioactivity profile and mode of action have not been fully investigated. Access to enough material is needed for these studies, but samples of these compounds are limited due to the minute amounts that can only be obtained by either large-scale cultivation of the organism that produces them or by total synthesis. Of all the amphidinolides known to date, only the targets of five of them (B1, H1, J, K, and X) have been examined and all have been found to interact with actin, a crucial cytoskeletal protein. This paper reviews what is currently known about actin-interacting amphidinolides, with a focus on the research of our group. Amphidinolides J and X are F-actin destabilizers, whereas Amphidinolides H1 and K stabilize actin filaments, likely via different mechanisms. More precise details of the interaction between amphidinolides and actin are missing.

## 1. Introduction

**Figure 1:**
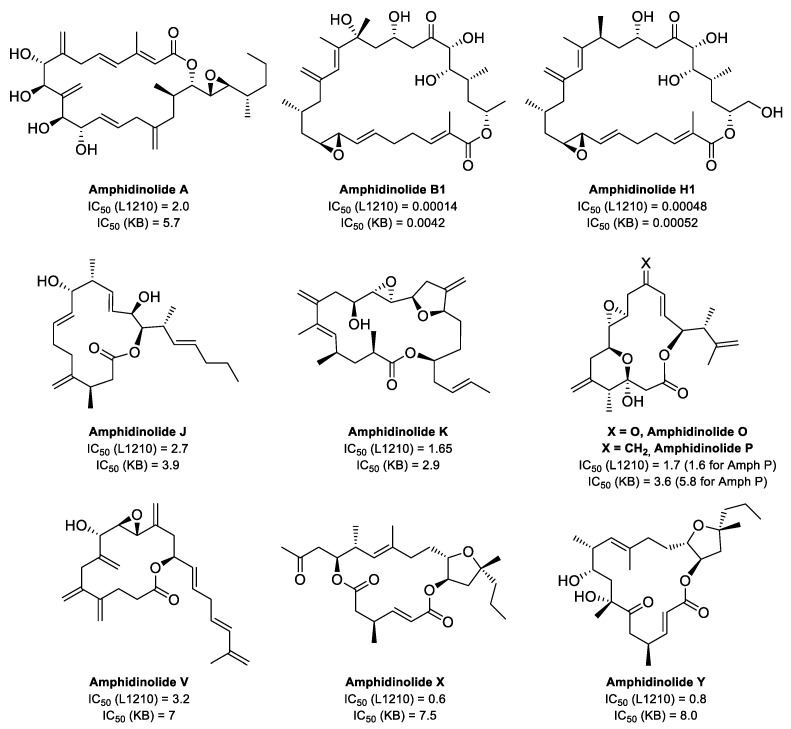
Structures of representative amphidinolides; IC_50_ in μg/mL; L1210, murine lymphoma cells; KB, human epidermoid carcinoma cells. Cytotoxicity data from ref. [2].

Amphidinolides, a family of marine macrolides produced by dinoflagellates of the genus *Amphidinium*, have attracted the interest of chemists worldwide because of their remarkable structures and bioactivities [1,2,3,4]. The first member of this family of compounds, Amphidinolide A, was isolated in 1986 [5]. Since then, more than 40 members have been reported (for relevant examples, see Figure 1). Although the cytotoxicities of medium-sized amphidinolides are only moderate, those of their larger counterparts can be in the nanomolar range. However, due to the scarcity of the natural compounds, very little is known about their mechanisms of action. Most amphidinolides have been obtained by Kobayashi in very low yields through large-scale cultivation in the lab of *Amphidinium* sp. [3]. Total synthesis also affords very small amounts of these compounds because their intricate structures, with many stereocenters, require complex multi-step syntheses with careful control of the absolute configuration. Despite this, in some cases enough samples have been available for testing and several amphidinolides have been shown to interact with actin. Synthesis has also provided some analogs of the natural compounds and several structure-activity studies have been published [6,7,8].

This review summarizes the current knowledge about the mechanisms of action of amphidinolides, with a special focus on the work of our research group. So far, there are only limited studies about the biological target of five different amphidinolides. Amphidinolide B1 was first reported to increase the activity of actomyosin, the actin–myosin complex [9]. In the following years, Amphidinolides J and X were shown to destabilize actin filaments [10], whereas Amphidinolides H1 [11,12] and K [8] are F-actin stabilizers. In particular, Amphidinolide H1 exerts its stabilizing effect by a novel mechanism, covalently binding to actin.

## 2. The Actin Cytoskeleton

**Figure 2:**
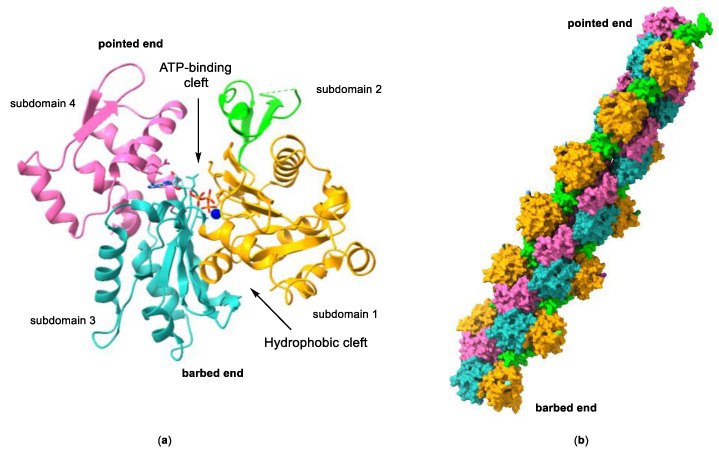
(**a**) Structure of G-actin showing the ATP-binding cleft and the hydrophobic cleft. Subdomains 1 to 4 are labelled and represented in different colors. The protein is shown as a ribbon representation and the bound nucleotide in a stick representation. Structure taken from ref. [16] (PDB accession code 3EL2). (**b**) Surface representation of a model of an actin filament. The subdomains for each actin monomer are color-coded as in (**a**). Structure taken from ref. [17] (PDB accession code 3G37).

Actin is a highly conserved family of proteins that plays a key role in the cytoskeleton of all eukaryotic cells, alongside microtubules and intermediate filaments. The actin monomer, also known as globular actin or G-actin, is a 43 kDa protein with four subdomains that form two binding sites. ATP and Mg^2+^ bind to a large cleft formed between subdomains 2 and 4, whereas the hydrophobic surface formed by subdomains 1 and 3 (barbed end) contains a smaller cleft, known as the hydrophobic cleft, that is an important binding site for actin-binding proteins (ABPs), and other molecules (Figure 2). Under physiological conditions, three G-actin monomers can spontaneously assemble head to tail (nucleation), initiating filament growth and forming long actin filaments (F-actin), composed of two parallel protofilaments twisted around each other generating a right-handed helix. The incorporation of more G-actin monomers can take place from both ends of the protofilament, although the rate of monomer incorporation is different at each end. The rate of growth at the pointed end, formed by subdomains 4 and 2, and also known as the minus end, is considerably slower than at the barbed end (or plus end). This asymmetric growth is attributed to the change of conformation that occurs in subdomain 2 as a result of ATP hydrolysis once G-actin is incorporated into the filament.

The dynamic polymerization/depolymerization equilibrium, essential in the regulation of many important cellular processes, is controlled not only by ATP hydrolysis but also by ions and many ABPs (for example, gelsolin and profilin) [13]. In vertebrates, three main isoforms of actin, α, β, and γ, can be found. α-actin is expressed only in muscle cells and, in association with the molecular motor myosin, takes part in muscle contraction. On the other hand, β- and γ-actin form the cytoskeleton of most cell types and are implicated in cell motility, cell division and cell shape, among others [14,15]. A precise control of the dynamics of actin polymerization is central for the correct functioning of the actin cytoskeleton and ABPs play an important role in this regard. Some proteins slow polymerization by binding to monomeric actin, inhibiting its addition to the growing filament and thus slowing filament growth (sequestering proteins). Others exert the same effect by binding to filament ends (capping proteins). In contrast, nucleation proteins favor polymerization. Other proteins break the filaments (cofilin, for example) and some proteins can have multiple roles.

The G-actin/F-actin equilibrium can also be affected by small molecules, and many natural compounds have been shown to exert their cytotoxic effect by disrupting the actin cytoskeleton dynamics [18,19,20,21,22]. Some of them, like Phalloidin, the gold standard F-actin marker, and Jasplakinolide, are already being used as probes to study cellular processes in which actin plays a key role [23]. Another attractive possibility is the use of these compounds as drugs. Indeed, actin and ABPs are key participants in cancer-related processes such as invasion and metastasis [24]. However, actin is ubiquitous in both healthy and cancer cells, so the disruption of actin polymerization by these potential drugs produces many undesirable side effects, making them too toxic for clinical use. So far, no actin-targeting drugs have reached the market. Despite their structural diversity, actin-binding natural products usually share some common features. Many are stereochemically complex large- or medium-sized macrolides sporting a side chain. These compounds can be broadly classified into two groups: those that inhibit filament formation (F-actin destabilizers or actin depolymerizing agents) and those that stabilize actin filaments (F-actin stabilizers or actin polymerizing agents).

**Figure 3:**
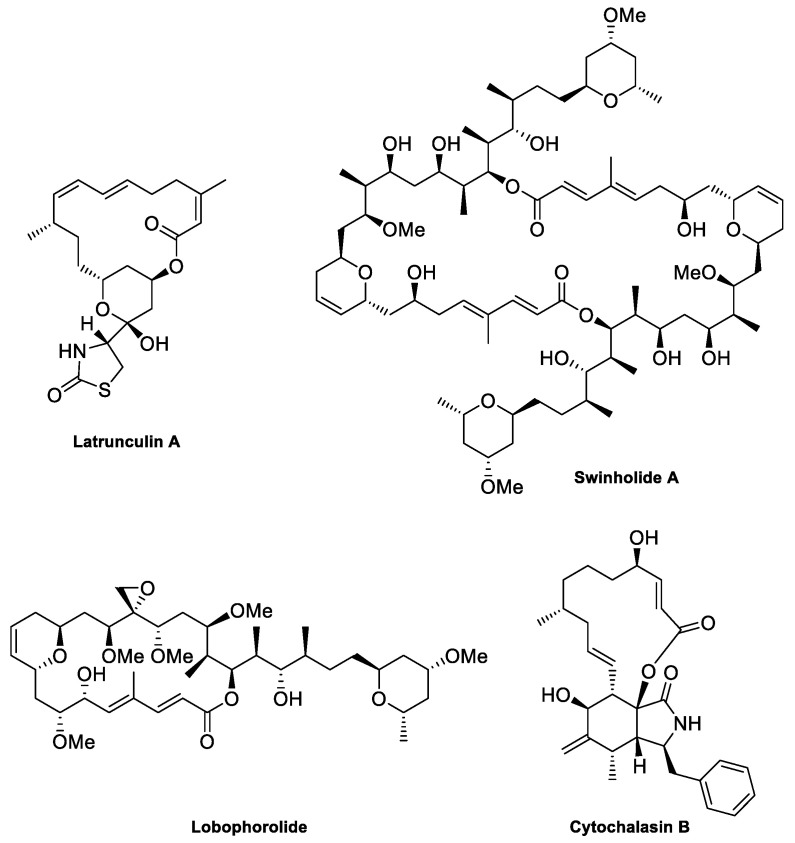
Structures of several F-actin-destabilizing macrolides.

F-actin destabilizers are compounds that prevent G-actin polymerization or that have the ability to disrupt or destabilize already formed actin filaments in cells. There are several mechanisms by which they can accomplish this. Some F-actin destabilizers bind to actin monomers, inhibiting their aggregation (G-actin sequestering), while others bind to the barbed end of F-actin, preventing further polymerization (capping of actin filaments), or actively break formed filaments by binding to them (severing of actin filaments). The latrunculins are known to inhibit actin filament formation by the sequestration of G-actin. Crystallographic studies have shown that the thiazolidinone ring of latrunculins binds to the ATP binding site of G-actin, an interaction stabilized by hydrophobic contacts of the macrocyclic ring with actin [25,26,27]. This causes allosteric changes in the monomer that hinder the interaction between G-actin monomers that leads to aggregation. More common are macrolides that prevent filament growth by binding to a large hydrophobic patch on the surface of actin formed by subdomains 1 and 3 (barbed end), between which the narrow hydrophobic cleft is found. At least seven different types of natural products are known to act in this way, for example, the swinholides, Lobophorolide or cytochalasins [28]. These compounds tend to have larger and more structurally diverse macrocyclic rings, and their side chains are long and stereochemically complex. The interaction of the large hydrophobic macrocyclic rings with different regions of this hydrophobic patch, depending on the compound, positions the side chains so that they can intercalate into the hydrophobic cleft. Because the hydrophobic patch is an important site for intermonomer contacts and the binding of ABPs, when a macrolide occupies it, filament growth is compromised. For example, Cytochalasin B (Figure 3) is thought to predominantly interact with actin in this way [29,30]. The compounds targeting either the ATP-binding site or the barbed-end binding site both act by sequestering G-actin and preventing its polymerization. However, some of the macrolides that target the hydrophobic patch on the barbed end also have the capacity to slow filament growth and/or to sever already-formed actin filaments. Because the hydrophobic patch is exposed on actin filaments and is thus accessible to macrolides, their binding to the barbed end of a filament slows its growth by capping. Additionally, upon binding of the macrolide to the filament, its side chain can orient in such a way as to be able to insert into the hydrophobic cleft, promoting conformational changes that disrupt intermonomer contacts; these are vital for filament integrity and can cause complete filament disruption. Many of the compounds that interact with the hydrophobic patch form a 1:1 complex with actin, although other stoichiometries have been unveiled. For example, Swinholide A, a 44-membered dimeric lactone, has been shown to form a 2:1 actin–macrolide complex in which each of the two side chains of the molecule binds to the hydrophobic cleft of two actin monomers, thus inhibiting F-actin formation by sequestering G-actin [31,32]. In contrast, Lobophorolide forms a quaternary complex with a 2:2 stoichiometry in which two macrolide molecules act together to stabilize an actin dimer [33].

**Figure 4:**
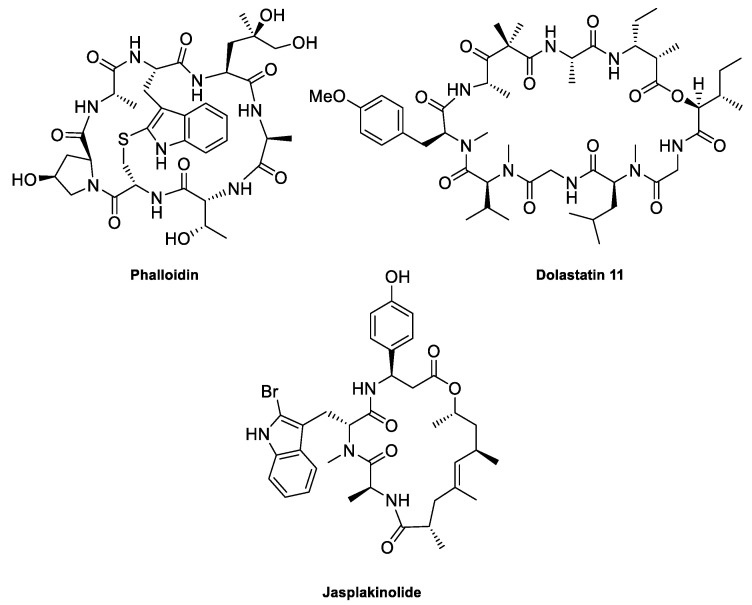
Structures of some F-actin-stabilizing natural product cyclopeptides.

F-actin-stabilizing compounds are molecules that either promote the polymerization of G-actin monomers or have the ability to enhance the stability of actin filaments in cells. Many of the natural products that stabilize F-actin bind several G-actin monomers simultaneously, stabilizing their interaction. This is the case, for example, for Phalloidin, which has been shown to interact with three different actin monomers via three different binding sites [30], and for Dolastatin 11, which interacts with two actin monomers [34] (Figure 4). Because the polymerization and stability of F-actin relies on cooperativity, these local interactions have effects far beyond the binding site of the macrolide.

## 3. Interaction of Amphidinolides B1 and H1 with Actin

The first hint that the biological target of certain amphidinolides could be actin was the discovery of Matsunaga et al., in 1999 [9], that Amphidinolide B1 [35,36] activates the interaction of actin and myosin, resulting in an increase in the ATPase activity of actomyosin, the actin–myosin complex, thus enhancing the contractions of myofilaments. No structural details of this interaction were reported.

**Figure 5:**
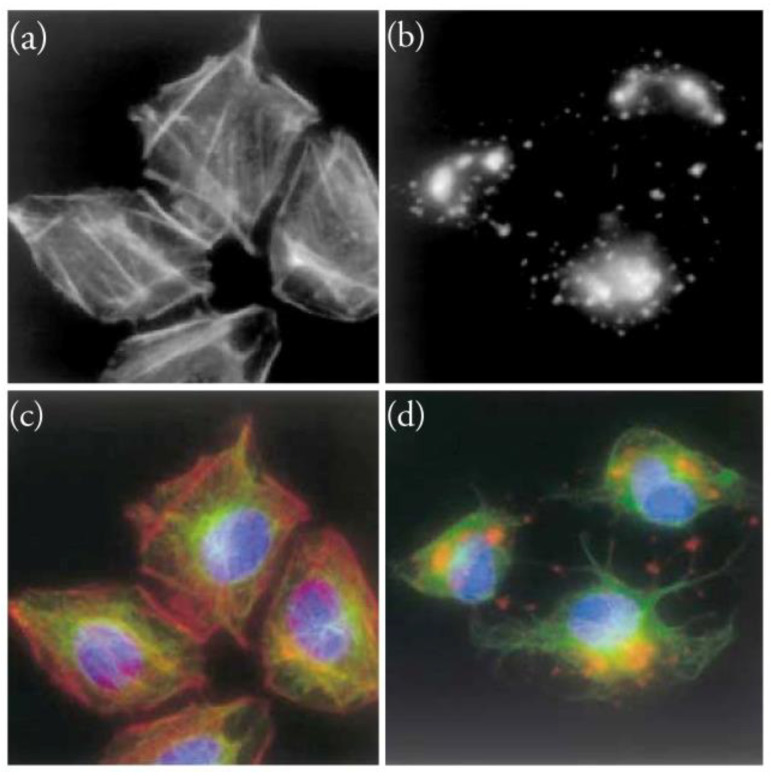
Effect of the treatment of 3Y1 cells with 30 nM Amphidinolide H1 for (**a**) 0 h; (**b**) 6 h. Merged images showing the effect of 30 nM Amphidinolide H1 on actin (red), tubulin (green) and DNA (blue) for (**c**) 0 h; (**d**) 6 h. Actin was stained with Rhodamine-Phalloidin. Tubulin was stained with anti-β-tubulin antibody. DNA was stained with Hoechst 33258. Reproduced from reference [11], with permission.

Five years later, Saito et al. [12] and Osada et al. [11] independently studied the effect of Amphidinolide H1 on actin. Both reports showed that Amphidinolide H1 [37], structurally very similar to Amphidinolide B1, stabilized F-actin via a different mechanism from that of previously known actin-stabilizers, that usually bind and thus bring together two or more G-actin monomers. Initially, actin was confirmed as the cellular target of Amphidinolide H1 when actin stress fibers of 3Y1 cells treated with this macrolide (30 nM) disappeared after 6 h, with only some aggregates remaining (Figure 5) [11]. Further experiments showed that this macrolide stimulates actin polymerization and stabilizes F-actin in vitro [11,12]. Finally, Amphidinolide H1 was shown by mass spectroscopy to covalently bind to actin through Tyr200 of actin subdomain 4, a residue that is close to subdomain 1 of the diagonally located G-actin monomer [11]. Although it could not be unambiguously proved, it is likely that Tyr200 of actin attacks the reactive vinyl epoxide moiety of Amphidinolide H1. In addition, Quartz Crystal Microbalance (QCM) experiments showed that Amphidinolide H1 can bind to both G-actin and F-actin. In contrast, Phalloidin, a well-known actin stabilizer, exclusively binds to F-actin. Some excellent total syntheses of Amphidinolides B1 [38,39,40] and H1 [40,41] were reported in the years following their isolation and biological study, but are not discussed in this work.

## 4. Total Syntheses of Amphidinolides X, J, and K Aimed at Elucidating Their Mechanisms of Action

Some years ago, we embarked on a project directed to the total synthesis of several members of the amphidinolide family. Apart from the synthetic challenge that such complex structures represent, our aim was also to gain insight into their mechanisms of action. At that point, nothing was known about the biological target of medium ring-sized amphidinolides, that are only moderately cytotoxic. Completion of the total syntheses of Amphidinolide X [42] and K [8] afforded a sufficient amount of these natural products to do a preliminary study of their interaction with actin [8,10]. At about the same time, the group of Cossy reported the total synthesis of Amphidinolide J [43], which was also submitted to biological evaluation [10]. In the following paragraphs, the total syntheses of Amphidinolides J, X, and K by the groups of Vilarrasa and Cossy will be reviewed, together with the experiments that led to the identification of actin as the probable biological target of these amphidinolides.

### 4.1. Total Synthesis of Amphidinolide X

**Scheme 1:**
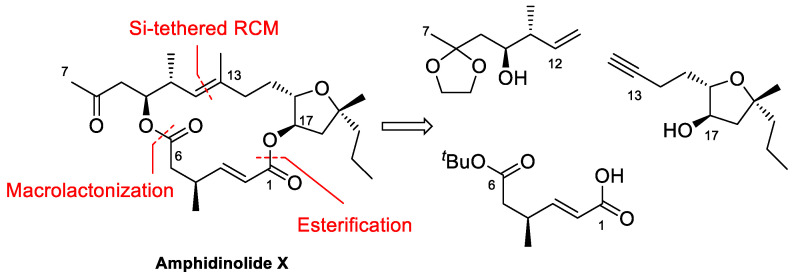
Retrosynthetic analysis of Amphidinolide X [42].

Amphidinolide X is a 16-membered amphidinolide with moderate cytotoxicity isolated by Kobayashi et al. in 2003 [44]. Its unusual nonsymmetric macrodiolide structure attracted the interest of research groups, so several total syntheses were soon reported [42,45,46,47,48]. In the retrosynthetic analysis of our group, shown in Scheme 1 [42], disconnection of the ester bonds and the C12–C13 alkene generates three fragments. Because the construction of the trisubstituted double bond via RCM turned out to be problematic, this bond was eventually formed via a Si-tethered cross-metathesis reaction.

**Scheme 2:**
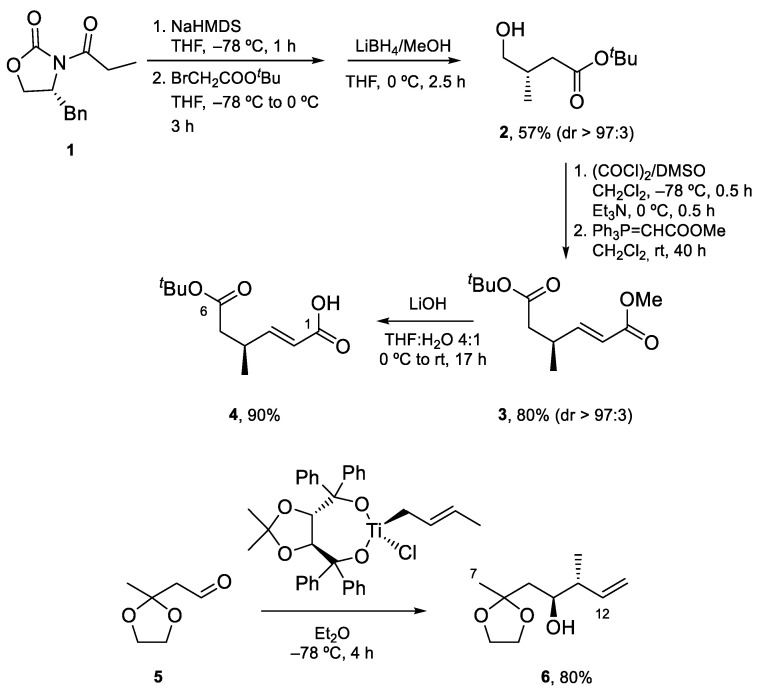
Synthesis of Fragment C1–C6 and Fragment C7–C12.

Fragment C1–C6 was prepared by alkylation of **1**, the *N*-propionyl derivative of one of Evans’ oxazolidinones, with *tert*-butyl bromoacetate, followed by the reductive removal of the auxiliary with LiBH_4_, Swern oxidation to the corresponding aldehyde, and Wittig reaction. Finally, selective saponification of the less bulky methyl ester afforded carboxylic acid **4**. Fragment C7–C12 was expediently prepared by asymmetric crotylation of aldehyde **5**, which is accessible in three steps from ethyl acetoacetate (Scheme 2).

**Scheme 3:**
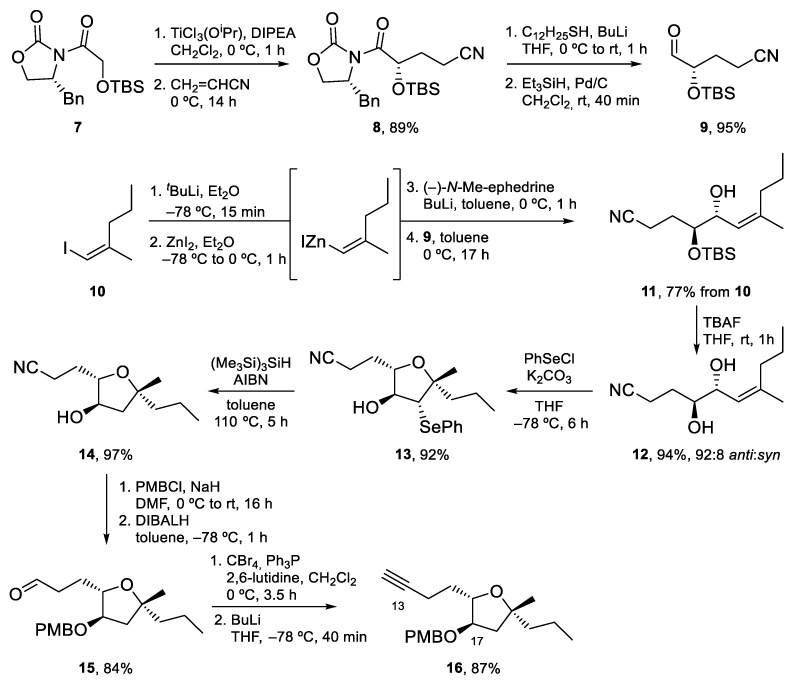
Synthesis of tetrahydrofuran **16**.

Synthesis of the tetrahydrofuran fragment started with the conjugate addition of the titanium enolate of **7** to acrylonitrile. Enantiopure aldehyde **9** was obtained after removal of the chiral auxiliary following Fukuyama’s method. Alkylation of this aldehyde with the alkenylzincate derived from alkene **10** afforded alcohol **11** as a 92:8 *anti*/*syn* mixture. After cleavage of the TBS protecting group under standard conditions, treatment with PhSeCl gave the desired tetrahydrofuran in excellent yield. Removal of the PhSe group using (Me_3_Si)_3_SiH (TTMS) afforded **14**, which was transformed into aldehyde **15** by protection of the hydroxy group as a PMB ether and DIBALH reduction. Finally, the Corey–Fuchs homologation of this aldehyde led to terminal alkyne **16** [49] (Scheme 3).

**Scheme 4:**
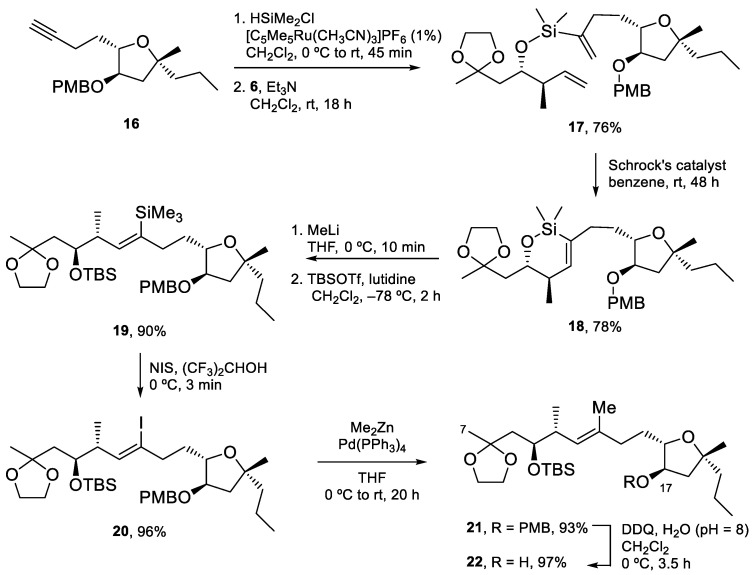
Synthesis of Fragment C7–C17.

Coupling of the fragments started with hydrosilylation of the triple bond of **16** using dimethylchlorosilane and the Trost catalyst, followed by a reaction of the resulting chlorosilane with alcohol **6** to yield silicon-tethered diene **17**. Ring-closing metathesis of this compound gave siloxane **18**. Conversion to the *E*-trisubstituted alkene took place by treatment of **18** with MeLi, to cleave the tether, followed by TBS protection, iododesilylation with NIS and Negishi coupling of the resulting vinyl iodide with Me_2_Zn. Finally, the PMB ether was cleaved with DDQ to afford **22** in excellent yield (Scheme 4).

**Scheme 5:**
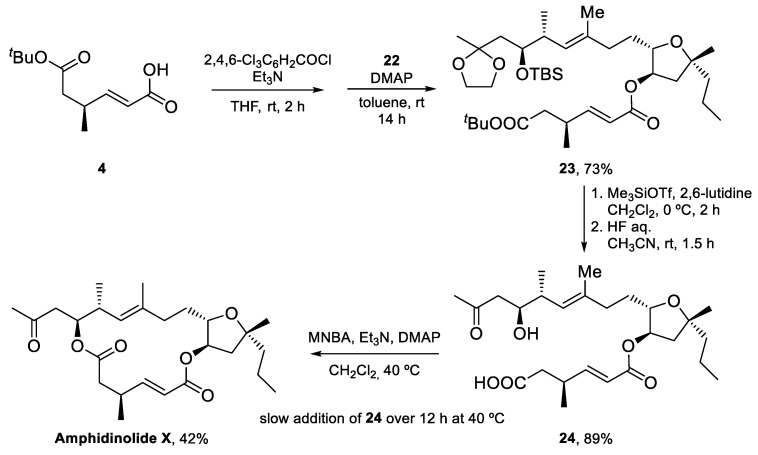
Completion of the synthesis of Amphidinolide X.

The synthesis of Amphidinolide X was completed by Yamaguchi esterification of acid **4** with tetrahydrofuran **22**, followed by global deprotection to yield *seco*-acid **24**, which was submitted to the conditions of the Shiina macrolactonization to furnish Amphidinolide X in 42% unoptimized yield [42] (Scheme 5).

The total synthesis of Amphidinolide X was thus achieved in 20 steps for the longest linear sequence and a 7% overall yield. A silicon-tethered metathesis reaction was employed to build the challenging trisubstituted double bond, which could not be prepared via a standard RCM reaction. The sample of Amphidinolide X obtained was used to study the potential interaction of this macrolide with actin, as described in Section 5.

### 4.2. Total Synthesis of Amphidinolide J

Amphidinolide J, a 15-membered macrolide first isolated by Kobayashi in 1993 [50], is another member of the amphidinolide family that has been shown to interact with actin. Like many medium-sized amphidinolides, it is only moderately cytotoxic but its complex structure, which includes six stereocenters, three *E*-disubstituted double bonds, and an exo-methylene group, makes it an attractive target for organic synthesis. The first total synthesis of this macrolide was reported in 1998 by Williams and Kissel [51], but it was the group of Cossy that, after obtaining enough sample of the macrolide by total synthesis [43], reported its interaction with actin [10].

**Scheme 6:**
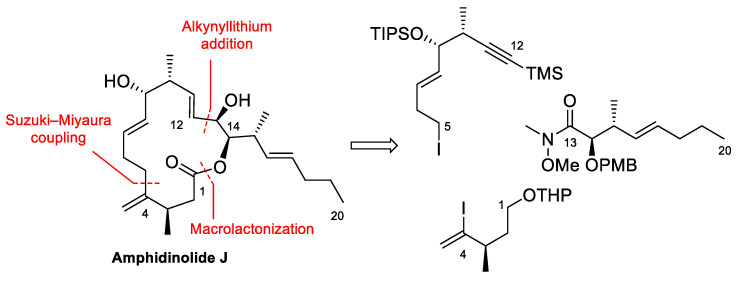
Retrosynthesis of Amphidinolide J [43].

The retrosynthesis designed by Cossy et al. to prepare Amphidinolide J disconnects the molecule into three fragments that are joined together via a Suzuki–Miyaura cross-coupling reaction, addition of an alkynyllithium to an aldehyde and formation of the macrolide ring by macrolactonization (Scheme 6).

**Scheme 7:**
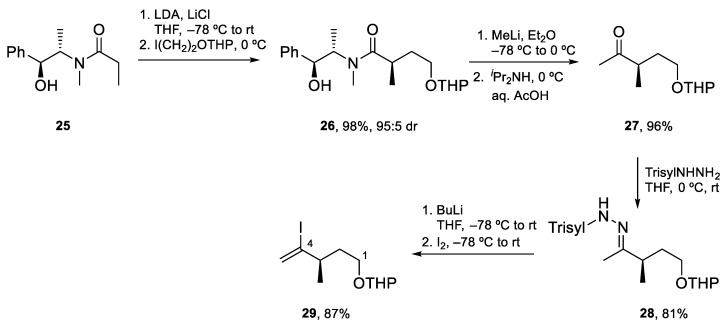
Preparation of Fragment C1–C4.

Fragment C1–C4 was prepared in four steps as shown in Scheme 7. The required stereocenter was introduced by Myers asymmetric alkylation of **25** with 2-iodoethanol THP ether (98%, ≥95:5 dr). Next, treatment of the resulting amide with MeLi afforded methyl ketone **27** which was transformed into the corresponding trisylhydrazone. Synthesis of the desired vinyl iodide was completed by Shapiro reaction and the trapping of the organolithium with iodine to obtain vinyl iodide **29** in excellent yield.

**Scheme 8:**
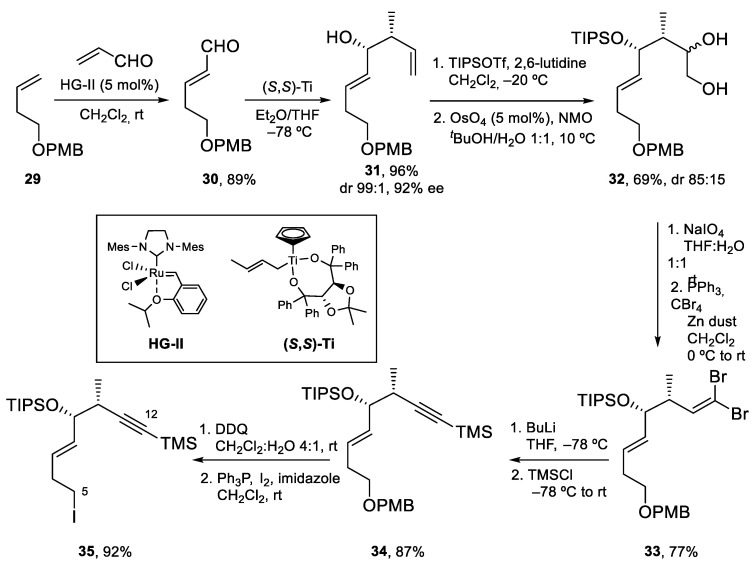
Synthesis of Fragment C5–C12.

The preparation of Fragment C5–C12 started by cross metathesis of PMB ether **29** with acrolein using the Hoveyda–Grubbs catalyst HG-II. The α,β-unsaturated aldehyde thus obtained (**30**) was submitted to a Duthaler–Hafner crotyltitanation that installed the two contiguous stereocenters with high diastereo- and enantioselectivity. The secondary alcohol was then protected as a bulky TIPS ether. This allowed the chemoselective dihydroxylation of the terminal alkene to 1,2-diol **32**, which was transformed into the corresponding aldehyde by oxidative cleavage with NaIO_4_. The sensitive aldehyde obtained was submitted to a Corey–Fuchs homologation to afford alkyne **34**. Finally, deprotection of the PMB ether with DDQ and transformation of the resulting alcohol into an iodide afforded **35**. In this way, Fragment C5–C12 was thus prepared from **29** in six steps and 36% overall yield (Scheme 8).

**Scheme 9:**
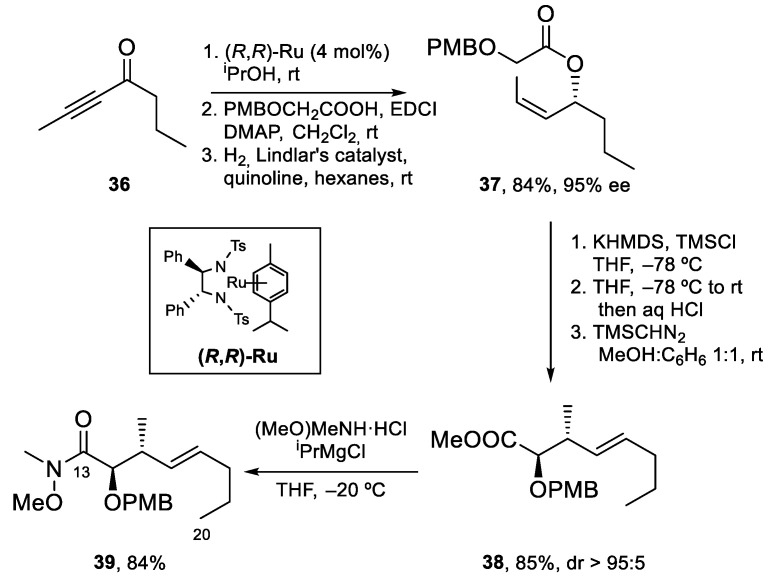
Synthesis of Fragment C13–C20 of Amphidinolide J.

For the preparation of Fragment C13–C20, alkynyl ketone **36** was enantioselectively reduced using the ruthenium catalyst (*R*,*R*)-Ru. Acylation of the resulting alcohol with PMB-protected 2-hydroxyacetic acid and hydrogenation of the alkyne to the *Z*-alkene using Lindlar’s catalyst afforded glycolate ester **37**, which was submitted to a stereoselective [3,3]-Claisen rearrangement through the intermediacy of a Z silyl ketene acetal. After hydrolysis and esterification with trimethylsilyldiazomethane, methyl ester **38** was obtained in excellent yield and diastereoselectivity (>95:5). Conversion of **38** to Weinreb amide **39** under standard conditions completed the synthesis of this fragment (Scheme 9).

**Scheme 10:**
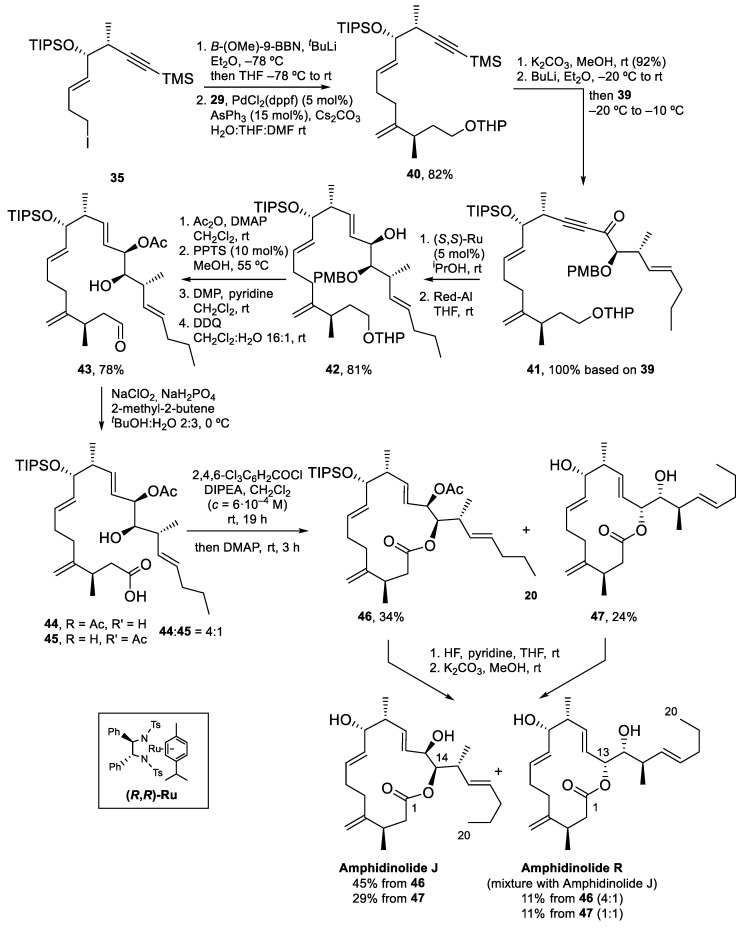
Completion of the synthesis of Amphidinolide J.

To attain the synthesis of Amphidinolide J (Scheme 10), iodide **35** was transformed into the corresponding 9-BBN borane and coupled with vinyl iodide **29** via a Pd-catalyzed Suzuki–Miyaura reaction, to obtain **40** in 82% yield. Next, deprotection of the alkyne with K_2_CO_3_, formation of the corresponding acetylide with BuLi and reaction with Weinreb amide **39** yielded **41**, which contains all the carbon atoms of Amphidinolide J. The acetylenic ketone present in **41** was diastereoselectively reduced to the alcohol (with reagent control, dr > 95:5) and the resulting propargylic alcohol was treated with Red-Al to furnish **42** in 81% yield. To prepare for the macrolactonization reaction, the C13 alcohol was protected as an acetate, the THP ether was deprotected and the resulting primary alcohol was oxidized to the corresponding aldehyde. The *seco*-acid was prepared by deprotection of the PMB ether with DDQ and Pinnick oxidation of the aldehyde. Unfortunately, during this last step, partial migration of the acetyl group to C14 was observed and a 4:1 inseparable mixture of two *seco*-acids, **44** and **45**, was isolated. Yamaguchi macrolactonization of this mixture afforded 15-membered macrolactone **46** and 14-membered macrolactone **47**, in 34 and 24% yield, respectively. Removal of the TIPS and acetyl protecting groups gave Amphidinolide J in 45% yield from **46**, together with a 4:1 mixture of Amphidinolides J and R (11%). From **47**, Amphidinolide J could be isolated as a pure compound in 29% yield (as before, a 4:1 mixture of Amphidinolides J and R was also isolated in 11% yield).

Amphidinolide J was thus prepared in 22 steps from **29** (longest linear sequence) and 4% overall yield. With the amount of Amphidinolide J obtained, studies towards the identification of its biological target were undertaken.

### 4.3. Total Synthesis of Amphidinolide K

**Scheme 11:**
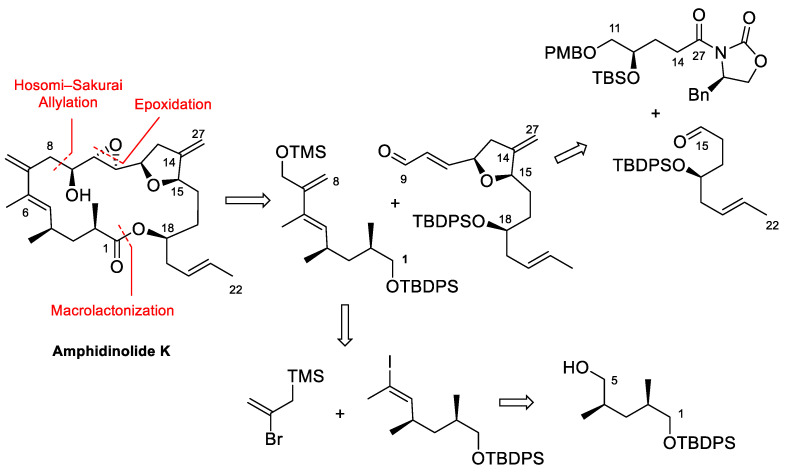
Vilarrasa’s retrosynthesis of Amphidinolide K.

Amphidinolide K (Figure 1 and Scheme 11) was first isolated by Kobayashi in 1993 [52]. It is a moderately cytotoxic 19-membered macrolide with a complex structure containing a tetrahydrofuran ring, an epoxide, a 1,3-diene and two exo-methylene groups, a feature common to many amphidinolides. Williams and Meyer described the first total synthesis of the enantiomer of this natural product in 2001 [53]. Later on, Lee et al. [54] and Vilarrasa et al. [8] also described synthetic approaches to this macrolide. Vilarrasa’s strategy disconnects this molecule into two major fragments as shown in Scheme 11. The tetrahydrofuran-containing fragment was prepared via a key aldol reaction between aldehyde C15–C22 and acyl oxazolidinone C11–C14/27, whereas allyl silane C1–C8 was ultimately built from alcohol C1–C5.

**Scheme 12:**
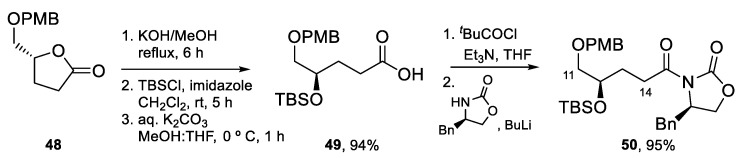
Preparation of acyl oxazolidinone **50**.

The synthesis of the tetrahydrofuran-containing fragment [55] started with the preparation of acyl oxazolidinone **50** from lactone **48** (Scheme 12), which was transformed into acid **49** by the opening of the lactone ring with KOH and protection of the resulting hydroxy group as a TBS ether. Following a standard protocol, acid **49** was transformed into **50**.

**Scheme 13:**
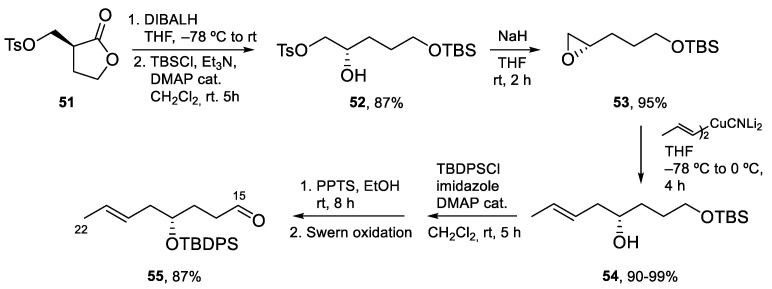
Synthesis of aldehyde C15–C22.

Aldehyde C15–C22 was prepared as shown in Scheme 13. The DIBALH reduction of lactone **51**, followed by the selective protection of the primary hydroxy group afforded alcohol **52**, that was converted into epoxide **53** by treatment with NaH. The requisite aldehyde **55** was obtained after addition of the cyanocuprate derived from (*E*)-1-propenyl-1-lithium to epoxide **53**, followed by the protection of the secondary hydroxy group as a TBDPS ether, selective deprotection of the TBS ether, and oxidation following the Swern protocol.

**Scheme 14:**
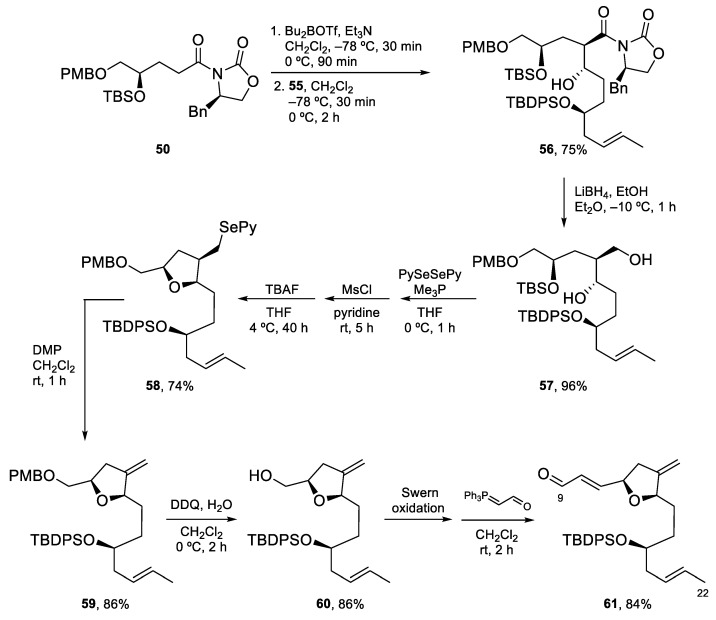
Synthesis of Fragment C9–C22.

The key aldol reaction between **50** and **55** (Scheme 14) required careful optimization to achieve a good yield of the aldol product. In the end, use of a dibutylboron enolate afforded the desired product in 75% yield. After the removal of the chiral auxiliary, conversion of the primary alcohol into a 2-pyridylselenyl derivative and activation of the secondary alcohol as a mesylate, the stage was set for the intramolecular S_N_2 reaction to form the tetrahydrofuran ring. Indeed, treatment with TBAF induced selective deprotection of the TBS ether with subsequent displacement of the mesylate group. Fragment synthesis was completed by elimination of the SePy group with Dess–Martin periodinane (DMP) at rt [56], PMB deprotection, and installation of the α,β-unsaturated aldehyde by Swern oxidation and Wittig reaction (Scheme 14).

**Scheme 15:**
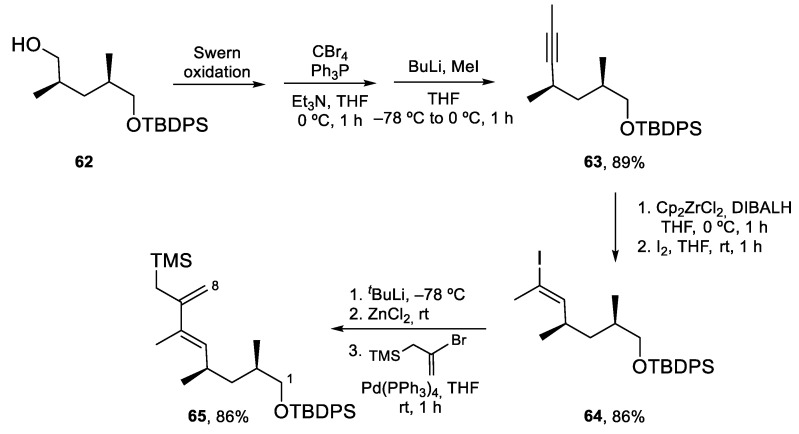
Preparation of allylsilane C1–C8.

The synthesis of allylsilane **65** (Scheme 15) started from known alcohol **62**, which was oxidized to the corresponding aldehyde and transformed into alkyne **63** following the standard Corey–Fuchs protocol. The (*E*)-vinyl iodide moiety was then installed by hydrozirconization/iodination. Allylsilane **65** was obtained by Negishi coupling of the organozinc compound derived from **64** with commercially available (2-bromoallyl)trimethylsilane in excellent yields.

**Scheme 16:**
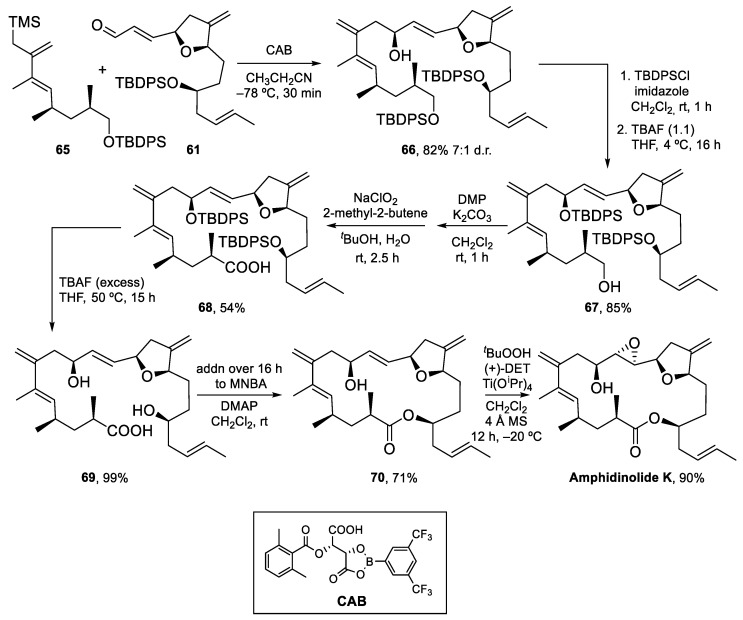
Completion of the synthesis of Amphidinolide K.

The key Hosomi–Sakurai allylation of aldehyde **61** was carried out with allylsilane **65** and the chiral acyloxyborane (CAB) catalyst developed by Yamamoto et al. (Scheme 16) [57]. With 1 equiv of CAB, the desired product **66** was isolated in 82% as a 7:1 inseparable mixture of diastereomers. Next, the protection of the secondary alcohol and selective deprotection of the primary TBDPS ether under carefully optimized conditions afforded primary alcohol **67**, which was oxidized to the carboxylic acid by treatment with DMP, followed by Pinnick oxidation. Carboxylic acid **68** was then fully deprotected, to obtain dihydroxy acid **69** in excellent yield. Macrolactonization of **69** under Shiina conditions afforded an excellent 71% of the desired macrolactone plus a 10% yield of what was assumed to be the C9-epimer, formed in the Hosomi–Sakurai allylation and which could be easily separated by column chromatography. Finally, Sharpless epoxidation of **70** gave Amphidinolide K in 90% yield (Scheme 16).

Amphidinolide K was thus prepared in 1% overall yield in 24 steps (longest linear sequence). Enough sample was obtained to study its possible interaction with actin, as described in the next section.

## 5. Interaction of Amphidinolides J, X, and K with Actin

**Figure 6:**
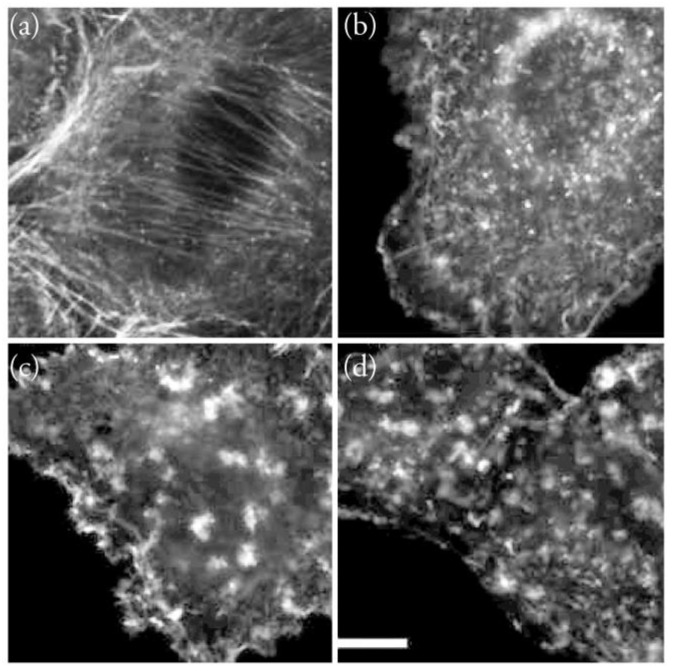
Effect of the incubation for 4 h of F-actin of A549 cells with (**a**) DMSO; (**b**) 250 μM Amphidinolide X; (**c**) 250 μM Amphidinolide J; (**d**) 2 μM Cytochalasin B. Actin was stained with Texas Red-X Phalloidin; DNA was stained with Hoechst 33342. Scale bar = 10 μm. Reproduced from reference [10], with permission.

**Figure 7:**
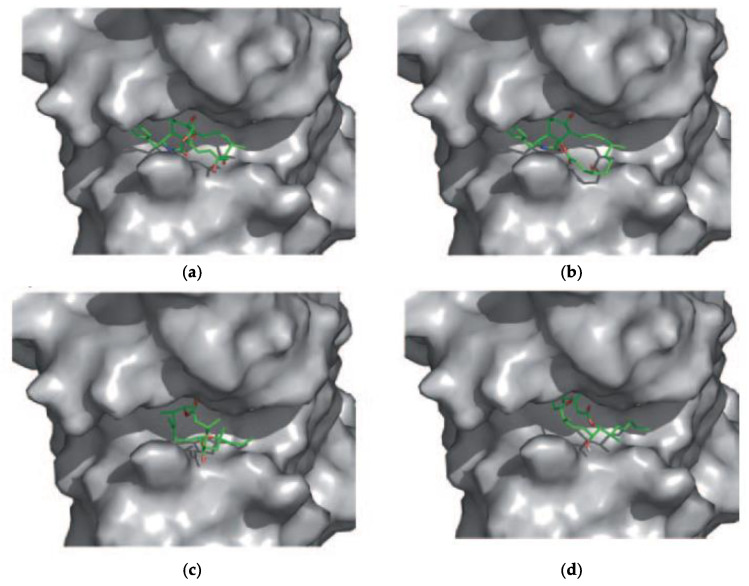
Complex of G-actin with (**a**) Cytochalasin D (PDB accession code 3EKS); AutoDock-generated complex of G-actin with (**b**) Cytochalasin D; (**c**) Amphidinolide X; (**d**) Amphidinolide J. The macrolides (ligands) are drawn in color (green for C; red for O; blue for N). Adapted from reference [10], with permission.

In 2011, the effects of Amphidinolides J and X on microtubules, intermediate filaments, and F-actin were first evaluated by immunofluorescent techniques [10]. When A549 non-small-cell lung carcinoma cells were incubated for 24 h with each of these macrolides, no changes in the tubulin or intermediate filament cytoskeleton were observed. However, changes in the shape and size of actin filaments were detected (Figure 6). Similar results were obtained in PtK2 cells. In vitro actin assembly and disassembly assays were then carried out in the presence of these amphidinolides. Both compounds inhibited actin assembly at 300 μM (20% inhibition for Amphidinolide X and 30% for Amphidinolide J). Cytochalasin B was used as a control and a 55% reduction of actin assembly was observed (at 25 μM). However, none of these macrolides had any effect on already formed actin filaments. The possible interaction of both amphidinolides with G-actin was also examined by means of docking simulations (AutoDock 3.0.5), which indicated that both amphidinolides fit well in the area of the hydrophobic patch between subdomains 1 and 3, where Cytochalasin B binds (Figure 7) [10]. All these data suggest that Amphidinolides X and J are relatively weak F-actin destabilizers that act by moderately inhibiting monomer addition to actin filaments due to their binding to the hydrophobic patch, as many other macrolides do.

Furthermore, preliminary experiments with Amphidinolide K showed that this macrolide does not interact with the microtubule cytoskeleton. However, when its effect on actin isolated from rabbit skeletal muscle was examined, with Phalloidin (an F-actin stabilizer) and Cytochalasin B (G-actin stabilizer) as references, it showed a strong stabilizing effect on F-actin (approximately 70% that of Phalloidin). More experiments are needed to understand the way in which Amphidinolide K stabilizes actin filaments.

## 6. Conclusions

Unraveling the mechanisms of action of bioactive compounds is essential for advancing our understanding of cellular processes, developing targeted therapies and uncovering disease mechanisms. Despite successful total syntheses of most amphidinolides known to date over the past two decades, providing access to samples for testing, our current understanding of their mechanisms of action remains limited. Data is available only for Amphidinolides B1, H1, J, X, and K, which have all been shown to interact with actin, albeit with different effects: Amphidinolides J and X moderately inhibit actin assembly, but do not affect already formed actin filaments, whereas Amphidinolides H1 and K stabilize F-actin. In the case of Amphidinolide H1, the strong stabilizing effect is due to the formation of a covalent complex with actin, involving the reactive vinyl epoxide moiety of the macrolide. Because of the structural similarity between Amphidinolides B1 and H1, their mode of action is likely similar. It is known that Amphidinolide B1 increases the ATPase activity of the actin–myosin complex, although no details of its effect on actin structure and dynamics have been reported. These disparate modes of action are to be expected because of the structural diversity of the members of this family of macrolides.

Studying the mechanisms of action of compounds that interact with actin is crucial to better understand the intricate interplay of processes in which actin plays a key role, helping us to gain insight into the regulation of actin dynamics and how perturbations in actin function can impact cellular behavior. These compounds could serve as powerful tools to probe and manipulate cytoskeletal organization; actin-interacting natural products such as Phalloidin and Jasplakinolide are already commonly employed in these studies. Additionally, because actin dysfunction is associated with various diseases, such as cancer metastasis, investigating the mechanisms by which compounds interact with actin can shed light on the underlying molecular basis of these diseases, and help identify key signaling pathways, molecular targets, and potential therapeutic strategies to counteract actin-related pathologies. In this way, valuable information for the development of drugs that modulate actin dynamics, aiding in the design of more effective and targeted therapies could be obtained.

## Data Availability

Data sharing not applicable. No new data were created or analyzed in this study.
